# Good learning environment of medical schools is an independent predictor for medical students’ study engagement

**DOI:** 10.3389/fmed.2024.1299805

**Published:** 2024-07-31

**Authors:** Runzhi Huang, Yuanan Li, Meiqiong Gong, Wei Zhang, Shuyuan Xian, Jieling Tang, Bingnan Lu, Yiting Yang, Minghao Jin, Weijin Qian, Zhenglin Liu, Haonan Ma, Xinru Wu, Huabin Yin, Xin Liu, Chongyou Zhang, Erbin Du, Qing Lin, Zongqiang Huang, Min Lin, Xiaonan Wang, Yue Wang, Wenfang Chen, Yifan Liu, Jie Zhang, Shizhao Ji

**Affiliations:** ^1^Department of Burn Surgery, The First Affiliated Hospital of Naval Medical University, Shanghai, China; ^2^Research Unit of Key Techniques for Treatment of Burns and Combined Burns and Trauma Injury, Chinese Academy of Medical Sciences, Shanghai, China; ^3^Shanghai Jiao Tong University School of Medicine, Shanghai, China; ^4^Office of Educational Administration, Shanghai University, Shanghai, China; ^5^Department of Orthopedics, Shanghai General Hospital, School of Medicine, Shanghai Jiaotong University, Shanghai, China; ^6^Department of Rheumatology and Immunology, Second Affiliated Hospital of Naval Medical University, Shanghai, China; ^7^Basic Medical College, Harbin Medical University, Heilongjiang, China; ^8^Frist Clinical Medical College, Mudanjiang Medical University, Mudanjiang, China; ^9^Department of Human Anatomy, Laboratory of Clinical Applied Anatomy, School of Basic Medical Sciences, Fujian Medical University, Fuzhou, China; ^10^Department of Orthopedics, The First Affiliated Hospital of Zhengzhou University, Zhengzhou, China; ^11^Mental Health Education and Consultation Center, Chongqing Medical University, Chongqing, China; ^12^Department of Epidemiology and Health Statistics, School of Public Health, Capital Medical University, Beijing, China; ^13^Department of Health Statistics, School of Public Health, Air Force Medical University, Xi’an, China; ^14^Faculty of Medicine, Jinggangshan University, Ji’an, China; ^15^Department of Gynecology, Shanghai First Maternity and Infant Hospital, Tongji University School of Medicine, Shanghai, China

**Keywords:** medical education, medical students, learning environment, study engagement, Utrecht Work Engagement Scale (UWES), nomogram

## Abstract

**Background:**

Study engagement is regarded important to medical students’ physical and mental wellbeing. However, the relationship between learning environment of medical schools and the study engagement of medical students was still unclear. This study was aimed to ascertain the positive effect of learning environment in study engagement.

**Methods:**

We collected 10,901 valid questionnaires from 12 medical universities in China, and UWES-S was utilized to assess the study engagement levels. Then Pearson Chi-Square test and Welch’s ANOVA test were conducted to find the relationship between study engagement and learning environment, and subgroup analysis was used to eradicate possible influence of confounding factors. After that, a multivariate analysis was performed to prove learning environment was an independent factor, and we constructed a nomogram as a predictive model.

**Results:**

With Pearson Chi-Square test (*p* < 0.001) and Welch’s ANOVA test (*p* < 0.001), it proved that a good learning environment contributed to a higher mean of UWES scores. Subgroup analysis also showed statistical significance (*p* < 0.001). In the multivariate analysis, we could find that, taking “Good” as reference, “Excellent” (OR = 0.329, 95%CI = 0.295–0.366, *p* < 0.001) learning environment was conducive to one’s study engagement, while “Common” (OR = 2.206, 95%CI = 1.989–2.446, *p* < 0.001), “Bad” (OR = 2.349, 95%CI = 1.597–3.454, *p* < 0.001), and “Terrible” (OR = 1.696, 95%CI = 1.015–2.834, *p* = 0.044) learning environment only resulted into relatively bad study engagement. Depending on the result, a nomogram was drawn, which had predictive discrimination and accuracy (AUC = 0.680).

**Conclusion:**

We concluded that learning environment of school was an independent factor of medical student’s study engagement. A higher level of learning environment of medical school came with a higher level of medical students’ study engagement. The nomogram could serve as a predictive reference for the educators and researchers.

## Introduction

Study engagement can be interpreted as having a positive, fulfilling, work-related state of mind (1). It was shown that study engagement could lead to less burnout and perceived stress (1). What’s more, study engagement is an important factor for the educational success and the development of a student (2). Previous research showed that study engagement had a positive effect on student’s ability of critical thinking (3). However, students who were disengaged in schoolwork had worse academic performance in school, were less likely to be employed and developed poorer psychological wellbeing (4), and they had greater chance of burnout (1). Medical students in China, who have to complete a large amount of medical trainings in a short period of time, are under more pressure than others (5, 6), especially during the period of COVID-19 (7). In a word, having a good study engagement is conducive to medical students’ academic performance, mental health and physical wellbeing. Previous research has suggested that student engagement can be enhanced by fostering positive relationships among peers and between students and faculty, improving students’ sense of competence, promoting agency and empowerment among students, and enhancing the perceived relevance of learning activities through meaningful engagement (8, 9). Besides, learning environment can also influence study engagement in medical students (10).

Learning environment is the physical, social and psychological contexts in which students learn (11). The existence of a positive learning environment has positive effect on students’ humanism, academic performance, and wellbeing (11–13). In the contrast, an unsupportive learning environment may result in burnout, worse quality of life, higher odds of emotional exhaustion, less empathy and poorer clinical performance in medical students (14–17). Previous studies have highlighted the importance of the learning environment for medical students, concluding that improvements are necessary across various aspects of this environment (11, 18, 19).

Since study engagement and learning environment are both proved to be connected with higher academic achievements and less odds of burnout, it was no surprise that they were corelated with each other. The relationship between learning environment of medical schools and study engagement of students is still in controversy. One previous research compared students’ engagement from three different campuses, which provided diverse learning environment, and found no statistically significant differences (20). In the contrary, study engagement was found to serve as the mediate factor between students’ perceptions of the school environment and their academic performances (21), and students’ perceptions of support from their peers and teachers could lead to greater learning engagement (22). Although the literature on learning environment as a protective factor of study engagement was notable, limitations still existed in the current study. First, the sample size of previous researches was relatively small (20–22). Second, the learning environment had not been analyzed as an independent factor of study engagement. Our study, which had a relatively big sample size, was aimed to prove the learning environment as an independent factor of study engagement, and then construct a predictive model, which included learning environment as a predictive variable.

China, one of the countries with the largest population in the world, boasts the world’s largest medical education system. In 2018, China had 420 undergraduate institutions offering medical education (23). However, it ranks among the 20 countries with the lowest ratio of medical schools per million inhabitants, resulting in an extremely strained healthcare resources in China, a situation far more severe compared to Western or other Asian countries (24).The high patient population combined with a relatively small number of doctors has created a demand for a large and robust healthcare workforce (25). Given these unique circumstances, which differ significantly from those of other countries, it is crucial to investigate the learning environment and study engagement of medical students in China.

Our previous study had conducted researches among 11 universities in China, finding out potential factor for learning environment and study engagement of medical schools (26, 27). This study delved deeper into understanding the impact of the learning environment on medical education. We hypothesized that the quality of the learning environment in medical schools serves as an independent predictor of medical students’ study engagement. To investigate this hypothesis, we conducted a study involving medical students from 12 universities in China. We examined whether variations in students’ perceptions of the learning environment correlated significantly with differences in study engagement. Additionally, if such variations were observed, we further explored the independent influence of the learning environment on study engagement using multivariate regression analysis. Finally, based on the results of the multivariate regression model, we developed a nomogram. Through this study, we aimed to shed light on the widely held belief that a positive medical learning environment fosters greater study engagement among medical students. Additionally, we sought to offer a predictive tool for assessing medical students’ study engagement.

## Materials and methods

### Sample source and data extraction

This study was approved by the Ethics Committee of the First Affiliated Hospital of the Second Naval Military Medical School (CHEC2023-284). Initially, we conducted a pilot study involving 20 students from the Naval Medical University, selected through stratified random sampling based on their grade level. Each student was coded according to their student number and integrated into a random number table corresponding to their grade. Subsequently, three undergraduate students and five graduate students were randomly selected from each grade to complete the questionnaire. The questionnaire, translated into Chinese from the original template, underwent modifications based on student feedback to enhance readability and coherence. All questions were transformed into positive declarative statements to align with the Likert scale, and certain terms were adjusted for easier comprehension in Chinese, such as replacing “student” with “classmate,” “school” with “medical school,” and “clinical” with “clinical field.” Additionally, predisposed terms were either removed or substituted with neutral equivalents, resulting in improved accuracy and fluency of the questionnaire. Subsequently, the revised questionnaire was deployed on the Wenjuanxing platform^1^ for data collection.

After that, the link to the standardized questionnaire was disseminated to relevant officials in the medical schools of the 12 universities mentioned earlier. Students were stratified by grade (from grade 1 to 5), and a random selection of students from one or two classes in each grade was invited to participate using stratified cluster sampling. Graduate medical students from seven universities also contributed to the study, while returning students were excluded. Prior to questionnaire distribution, all participating students were briefed on the study’s objectives and assured of the anonymity of their responses. A total of 12,600 questionnaires were distributed across the 12 universities, with 11,265 responses received. Following exclusion of incomplete or inaccurately filled questionnaires, 10,901 valid responses were retained for subsequent analysis.

### Instrument to measure students’ engagement and learning environment

The UWES-Student (UWES-S) was developed by Schaufeli et al. with college students as participants (28). This scale has been validated in Chinese college students as well (29). The scale’s contents, presented in Supplementary Table S1 (English version), consist of three dimensions (Vigor, Dedication, and Absorption) with a total of 16 items. Respondents rated all items on a 7-point Likert scale ranging from 1 (never) to 7 (always), with higher scores indicating higher levels of emotional engagement. In our study, a good internal reliability (total Cronbach’s α: 0.885) and structure validity (KMO value: 0.927, *p* < 0.001) was shown.

In our study, there were two different methods to assess the learning environment. In Step 1, respondents were asked to respond to the question, “What is the overall learning environment of your school?” They then categorized their school’s learning environment as “terrible,” “bad,” “common,” “excellent,” or “good.” In Step 2, we conducted a validation analysis using the Johns Hopkins Learning Environment Scale (JHLES) to assess medical students’ perceptions of their learning environment (30). The scale’s contents, presented in Supplementary Table S2 (English version), comprising seven subscales: Community of Peers, Faculty Relationships, Academic Climate, Meaningful Engagement, Mentoring, Inclusion and Safety, and Physical Space. In total, the scale includes 28 items. Each item was assessed using a five-point Likert response scale, ranging from strongly disagree (1) to strongly agree (5). Higher scores indicated a more positive perception of the learning environment.

### Step 1: global assessment of the learning environment

The exposure factor in our study was learning environment of the school. All the students were divided into two groups according to the calculated median value of their UWES scores. To find out the relationship between the exposure factor and UWES category and scores, we conducted Pearson Chi-Square test and Welch’s ANOVA test, respectively. The results were showed in the box plot and scatter diagram, respectively. Moreover, to exclude the possible influence of the certain confounding factors (gender, age) on those tests, subgroup tests were also conducted.

We then performed a multivariate regression analysis to prove learning engagement was an independent factor of study engagement. The multivariate regression analysis included 7 variables (age, gender, ethnicity, major, grade, native place, learning environment of your schools), and the odds ratio (OR) and *p* values were listed in a table. At last, a nomogram was constructed depending on the result of this multivariate regression model, and we used receiver operating characteristic (ROC), calibration curves and decision curve analysis (DCA) to diagnose this model.

### Step 2: validation with JHLES

In order to validate our result, the univariate linear regression analysis was conducted between JHLES scores and UWES scores among various subgroups (gender, age, ethnicity, major, learning environment of your schools, grade, and native place).

### Quantitative analysis instruments

In our study, categorical variables were presented as number (percentage), while continuous variables were represented as mean (standard deviation) or median (interquartile range). Parametric tests were employed for group means when variables exhibited a normal distribution and homogeneity of variance, including the Chi-Square test and ANOVA. Non-parametric tests were employed for group medians when variables did not adhere to a normal distribution or homogeneity of variance. Additionally, multivariate regression analysis was performed to validate that the learning environment was an independent factor influencing study engagement. Statistical significance was set as a two-sided *p* < 0.05. We performed the analytic processes by using R version 4.2.2 (Institute for Statistics and Mathematics, Vienna, Austria) and SPSS20.0 (IBM, New York, United States).

## Results

### Sample characteristics

**Figure 1:**
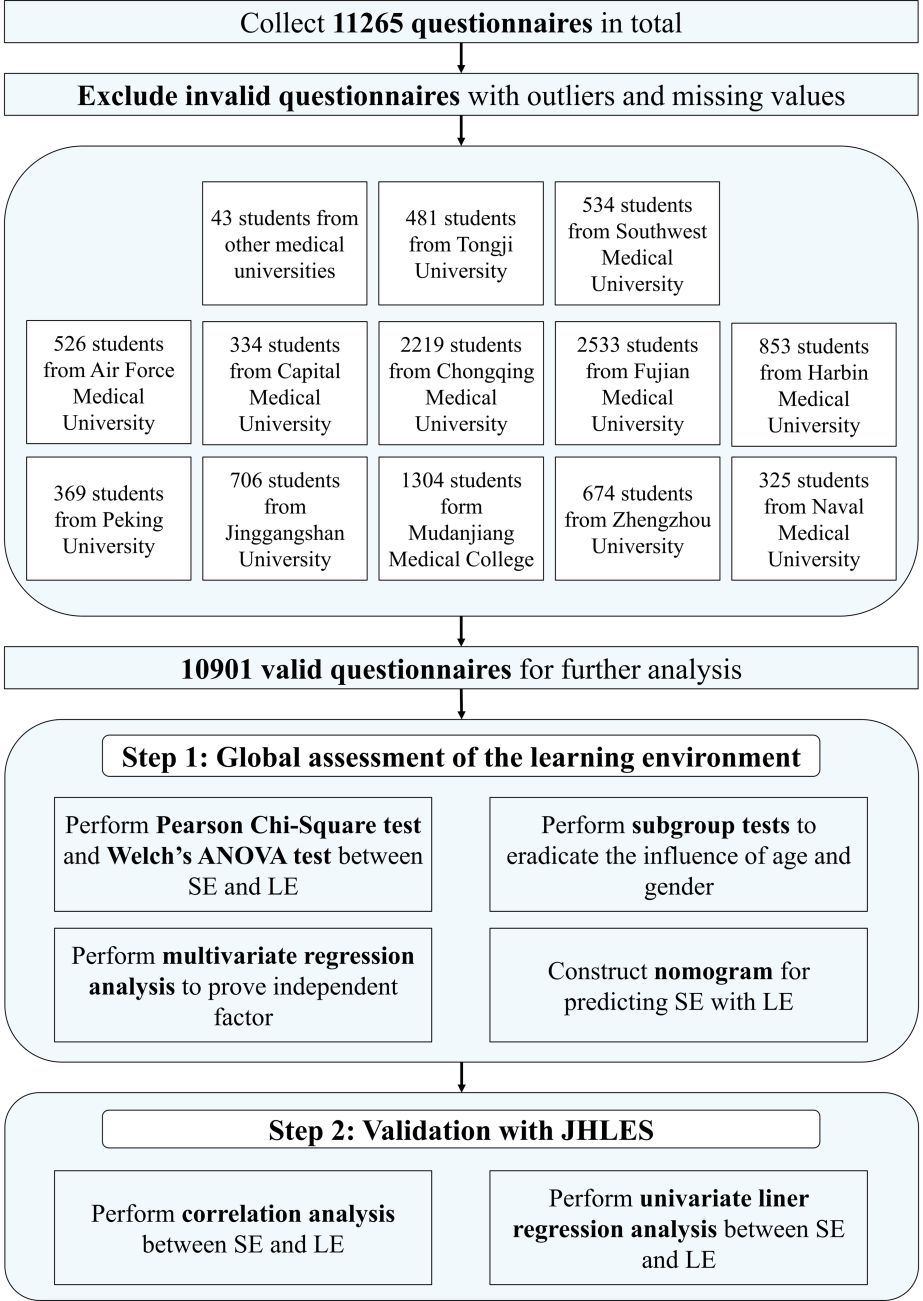
Flowchart of the study. LE, Learning Environment; SE, Study Engagement.

After distributing and collecting questionnaires, we had received 11,265 questionnaires to perform the study, and, after eliminating the invalid ones, a total of 10.901 samples were used to conduct further analysis (Figure 1). For the sake of visualizing the results, we integrated our data in Table 1.

**Table 1:** Characteristics of 10,901 students.

Variables	Number (percentage)
Gender	
Male	4,370 (40.09)
Female	6,531 (59.91)
Age	
16–20	5,868 (53.83)
21–25	4,825 (44.26)
26–40	208 (1.91)
University category	
211 project universities	692 (6.35)
985 project universities	853 (7.82)
Military University	851 (7.81)
Non 985/211 project universities	720 (6.60)
The first batches of medical universities	6,473 (59.38)
The second batches of medical universities	1,312 (12.04)
Universities	
Air Force Medical University	526 (4.83)
Capital Medical University	334 (3.06)
Chongqing Medical University	2,219 (20.36)
Fujian Medical University	2,533 (23.24)
Harbin Medical University	853 (7.82)
Jinggangshan University	706 (6.48)
Mudanjiang Medical College	1,304 (11.96)
Naval Medical University	325 (2.98)
Others	43 (0.39)
Peking University	369 (3.39)
Southwest Medical University	534 (4.90)
Tongji University	481 (4.41)
Zhengzhou University	674 (6.18)
Major	
Clinical medicine	8,668 (79.52)
Nursing	572 (5.25)
Preventive medicine	698 (6.40)
Preclinical medicine	658 (6.04)
Stomatology	305 (2.80)
Ethnicity	
Ethnic Han	10,190 (93.48)
Minority	711 (6.52)
Only child	
Yes	4,761 (43.67)
No	6,140 (56.33)
Grade	
Grade 1	3,800 (34.86)
Grade 2	2043 (18.74)
Grade 3	1,666 (15.28)
Grade 4	1869 (17.15)
Grade 5	1,298 (11.91)
Graduate	225 (2.06)
Native place	
Country	2,562 (23.50)
Municipality	1,535 (14.08)
Prefecture city	2063 (18.92)
Provincial capital	1,127 (10.34)
Town	1,196 (10.97)
Village	2,418 (22.18)
Educational system	
Eight-year	1,305 (11.97)
Five-year	7,621 (69.91)
Other	1,695 (15.55)
Seven-year	280 (2.57)
GPA	
Top 5%	815 (7.48)
5–20%	2,509 (23.02)
20–50%	3,844 (35.26)
50–80%	2,687 (24.65)
80–100%	1,046 (9.60)
Father’s educational level	
Bachelor degree	1,292 (11.85)
Graduate degree	251 (2.30)
Junior college	1,141 (10.47)
Junior high school	3,800 (34.86)
Preliminary school	1794 (16.46)
Senior high school	2,623 (24.06)
Father’s occupation	
Civil servant	1,083 (9.93)
Company employee	1,093 (10.03)
Freelance work	2,112 (19.37)
Individual household	1,092 (10.02)
Professional/Technical	1,150 (10.55)
Worker/Peasant	4,371 (40.10)
Mother’s educational level	
Bachelor degree	959 (8.80)
Graduate degree	174 (1.60)
Junior college	1,017 (9.33)
Junior high school	3,322 (30.47)
Preliminary school	3,180 (29.17)
Senior high school	2,249 (20.63)
Mother’s occupation	
Civil servant	634 (5.82)
Company employee	1,250 (11.47)
Freelance work	2,892 (26.53)
Individual household	791 (7.26)
Professional/Technical	1,363 (12.50)
Worker/Peasant	3,971 (36.43)
Learning environment	
Terrible	63 (0.58)
Bad	125 (1.15)
Common	2,284 (20.95)
Excellent	2,381 (21.84)
Good	6,048 (55.48)
Doctor-patient relationship in your hospitals	
Terrible	46 (0.42)
Bad	121 (1.11)
Common	2,812 (25.80)
Excellent	1732 (15.89)
Good	6,190 (56.78)
Interests of medicine	
Common	2,654 (24.35)
Extremely interested	1872 (17.17)
Extremely uninterested	65 (0.60)
Interested	6,145 (56.37)
Uninterested	165 (1.51)
Kolb learning experience	
Accommodating	3,695 (33.90)
Assimilating	3,213 (29.47)
Converging	1766 (16.20)
Diverging	2,227 (20.43)
UWES category	
High (≥72)	5,572 (51.11)
Low (<72)	5,329 (48.89)

GPA, grade point average; UWES, Utrecht Work Engagement Scale.

Females (59.91%) participated more in our study than males (40.09%). Most of the students were in their 16–20 (53.83%) or 21–25 (44.26%). More than half of the students were in the First Batches of Medical Universities (59.38%). More than three quarters of the students majored in clinical medicine (79.52%). Ethnic Han (93.48%) was the ethnicity of the vast majority of the students. Only-child students (43.67%) were relatively equal to not-only-child students (56.33%) in the number. Grade 1 students (34.86%) took a large part, which was followed by Grade 2 (18.74%) and Grade 4 (17.15%). Many students derived from country (23.50%) and village (22.18%). About three quarters of students were involved in the 5-year (69.91%) educational system. The parents of the students tended to have a low educational degree. Most of the students felt that they had a good learning environment (56.78% is good, 15.89% is excellent). 51.11% of students were categorized into high UWES category, while the rest of them (48.89%) had low UWES scores.

### Step 1: global assessment of the learning environment

#### Pearson Chi-Square test and Welch’s ANOVA test

**Figure 2:**
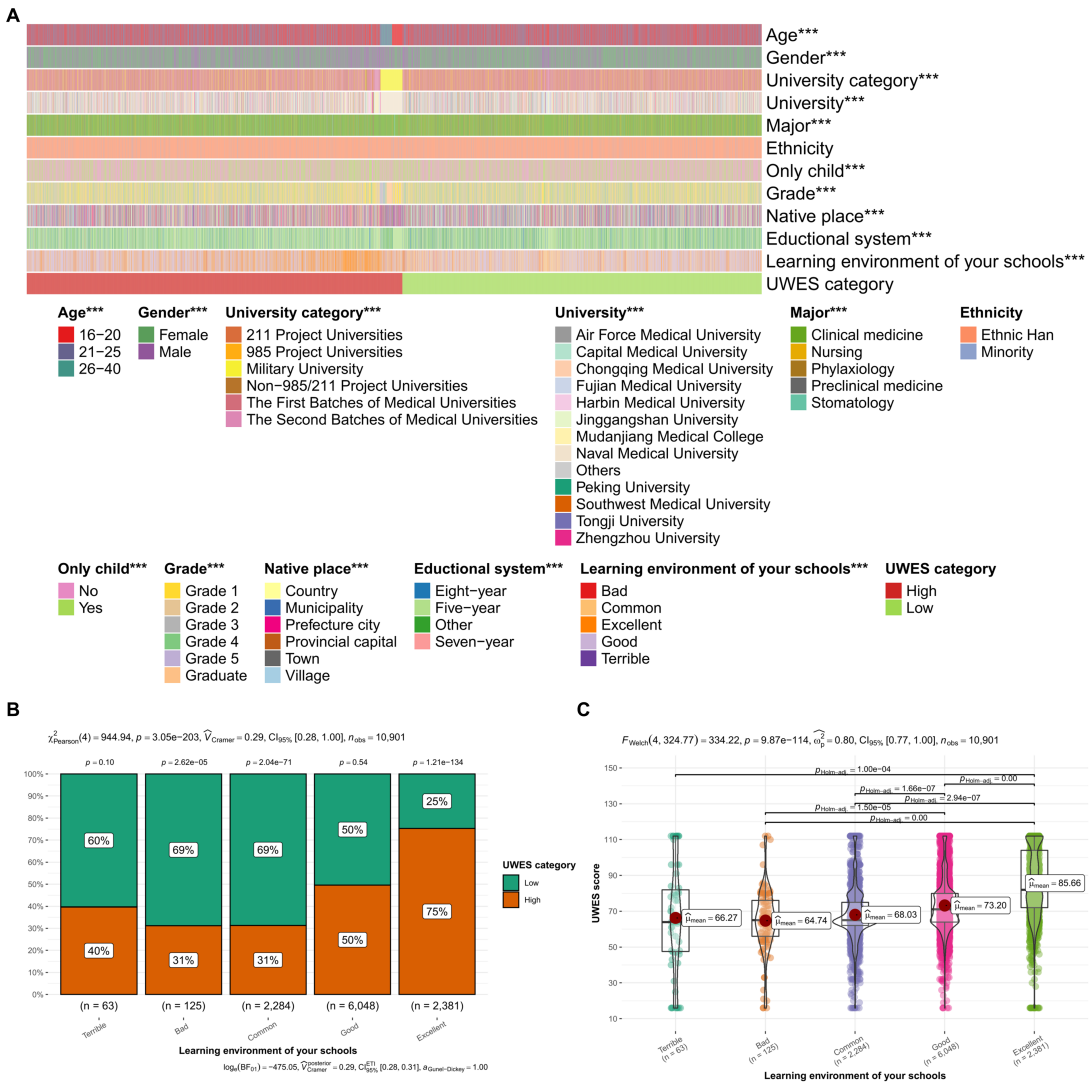
Pearson Chi-Square test and Welch’s ANOVA test. **(A)** Heatmap of the result of analysis. **(B)** The box plot of Pearson Chi-Square test. **(C)** The scatter diagram of Welch’s ANOVA test were further constructed, and both of them indicated that a better learning environment contributed to a better UWES score with *p* < 0.001. The Chi-square value was 𝜒^2^(4) = 944.94, which indicated a very strong association. The *F*-value of Welch’s ANOVA test was Fwelch (4,324.77) = 334.22, which shows a highly significant difference between the groups. *p < 0.05, **p < 0.01, ***p < 0.001; UWES, Utrecht Work Engagement Scale; GPA, Grade Point Average.

After collecting the data, we divided UWES scores into two groups depending on the calculated median value: one was equal and greater than 72, and the other was less than 72. We first performed Pearson Chi-Square test and Welch’s ANOVA test to prove that learning environment, as an influencing factor of UWES, was statically significant (Figure 2A). Then, to investigate the correlation between learning environment in your schools and UWES category, we performed the Pearson Chi-Square test. Besides, a Welch’s ANOVA test was conducted in study the relationship between learning environment in your schools and UWES scores.

In the result of Pearson Chi-Square test (Figure 2B), it can be observed that it was statistically significant [𝜒^2^(4) = 944.94, *p* < 0.001] when it comes to different ratio of high and low UWES categories in different learning environment. In the subgroups of “Terrible,” “Bad,” and “Common,” students with low UWES category seemed to be the majority (40, 31, 31%, respectively). In the subgroup of “Good,” the number of those who were in low UWES category were equal to that of students in high UWES category. And in the subgroup of “Excellent,” students in low UWES category only accounted for a quarter (25%). A elementary conclusion can be drawn from this result that students in better learning environment led to being in high UWES category.

The result of Welch’s ANOVA test (Figure 2C), which was demonstrated in a scatter plot, could prove to be statistically significant [Fwelch (4,324.77) = 334.22, *p* < 0.001]. It could be concluded that a good learning environment contributed to a higher mean of UWES scores.

**Figure 3:**
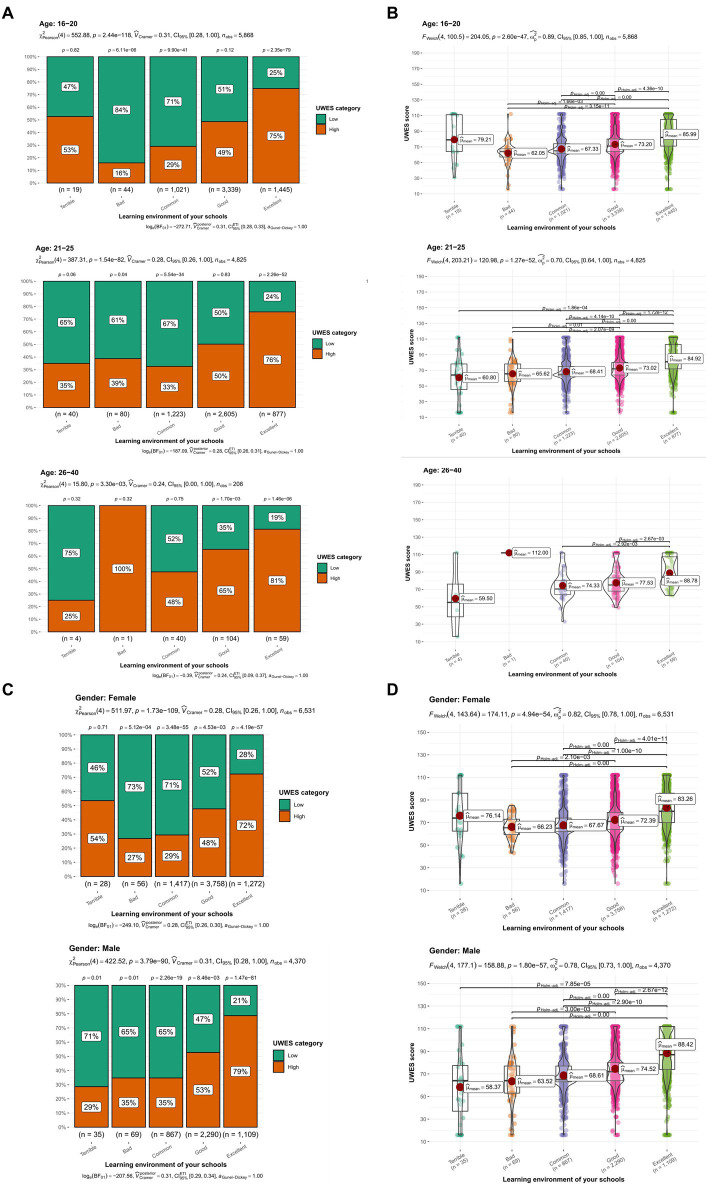
Subgroup analysis. **(A)** The box plot in different age subgroups. **(B)** The scatter plot in different age subgroups. **(C)** The box plot in different gender subgroups. **(D)** The scatter plot in different gender subgroups. All the subgroups, except “Age 26–40,” showed statistical significance, implying that learning environment was an influencing factor of study engagement after excluding confounding factors. UWES, Utrecht Work Engagement Scale.

What’s more, we further validated the conclusion with subgroup analysis. Dividing students into three subgroups depending on age and two subgroups depending on gender, we then performed Person Chi-Square test and Welch’s ANOVA test, respectively (Figures 3A–D). All the subgroup analyses showed statistical significance (*p* < 0.001) except the subgroup “Age 26–40,” which might be attributed to the small sample size of it. The subgroup analyses excluded possible influence of the confounding factors (gender, age) on the tests we conducted above.

#### Multivariate analysis

Then, we concluded 7 variables (age, gender, ethnicity, major, grade, native place, learning environment of your schools) in a multivariate regression analysis to prove that learning environment was an independent influencing factor of UWES scores, and the results, including the odds ratio and confidence intervals, were listed in Table 2. It should be noted that higher OR in this model meant lower UWES scores, thus worse study engagement.

**Table 2:** Multivariate logistic regression analysis of UWES scores.

Variables	UWES scores	
	OR (95% CI)	*P*-value
Age		
16–20	Reference	
21–25	0.911 (0.790, 1.051)	0.202
26–40	0.645 (0.440, 0.945)	0.024*
Gender		
Female	Reference	
Male	0.836 (0.770, 0.908)	< 0.001*
Ethnicity		
Ethnic Han	Reference	
Minority	1.094 (0.931, 1.286)	0.276
Major		
Clinical medicine	Reference	
Nursing	1.604 (1.330, 1.933)	< 0.001*
Preventive medicine	1.389 (1.178, 1.639)	< 0.001*
Preclinical medicine	1.069 (0.905, 1.264)	0.431
Stomatology	0.748 (0.586, 0.956)	0.02*
Grade		
Grade 1	Reference	
Grade 2	1.132 (1.007, 1.271)	0.037*
Grade 3	1.226 (1.050, 1.432)	0.01*
Grade 4	1.169 (0.976, 1.399)	0.09
Grade 5	0.881 (0.726, 1.071)	0.204
Graduate	0.983 (0.684, 1.413)	0.928
Native place		
Country	Reference	
Municipality	1.078 (0.942, 1.233)	0.274
Prefecture city	0.749 (0.662, 0.847)	< 0.001*
Provincial capital	0.765 (0.659, 0.887)	< 0.001*
Town	0.975 (0.844, 1.127)	0.734
Village	0.918 (0.817, 1.033)	0.155
Learning environment of your schools		
Good	Reference	
Bad	2.349 (1.597, 3.454)	< 0.001*
Common	2.206 (1.989, 2.446)	< 0.001*
Excellent	0.329 (0.295, 0.366)	< 0.001*
Terrible	1.696 (1.015, 2.834)	0.044*

OR, odds ratio; CI, confidence interval; GPA, grade point average; UWES, Utrecht Work Engagement Scale. *P < 0.05.

In the result of multivariate logistic regression analysis, it was indicated that male students (OR = 0.836, 95%CI = 0.770–0.908, *p* < 0.001) were less likely to be more engaged in study than female counterparts. As for age, 26–40 (OR = 0.645, 95%CI = 0.440–0.945, *p* = 0.024) was the age period in which students had better study engagement. Students majoring nursing (OR = 1.604, 95%CI = 1.330–1.933, *p* < 0.001) and preventive medicine (OR = 1.389, 95%CI = 1.178–1.639, *p* < 0.001) were two factors of low UWES probability, and stomatology (OR = 0.748, 95%CI = 0.586–0.956, *p* = 0.02) students enjoyed good study engagement. When it comes to Grade, Grade 3 (OR = 1.226, 95%CI = 1.050–1.432, *p* = 0.01) and Grade 2 (OR = 1.132, 95%CI = 1.007–1.271, *p* = 0.037) students indicated low study engagement. What’s more, students from provincial capital (OR = 0.765, 95%CI = 0.659–0.887, *p* < 0.001) and prefecture city (OR = 0.749, 95%CI = 0.662–0.847, *p* < 0.001) seemed to be more engaged in study than other ones. For learning environment of your schools, those who deemed their learning environment as bad (OR = 2.349, 95%CI = 1.597–3.454, *p* < 0.001), common (OR = 2.206, 95%CI = 1.989–2.446, *p* < 0.001) and terrible (OR = 1.696, 95%CI = 1.015–2.834, *p* = 0.044) were under highest risk of bad study engagement, and those who thought their learning environment was excellent (OR = 0.329, 95%CI = 0.295–0.366, *p* < 0.001) had better study engagement.

**Figure 4:**
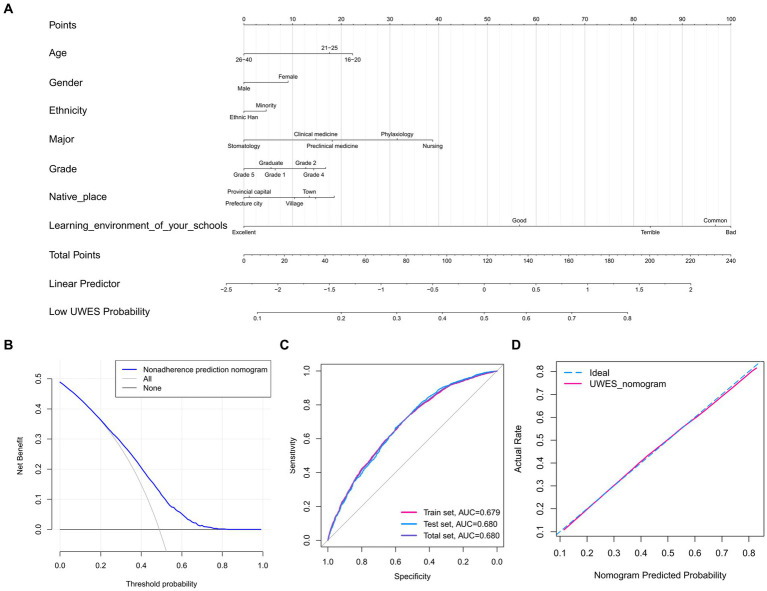
Nomogram and nomogram validation. **(A)** Nomogram. It provided a prediction of the low UWES probability with one’s learning environment. **(B)** DCA of the nomogram. When the threshold probability was >0.2, the medical students had higher net benefits. **(C)** ROC of the nomogram. It was indicated by the ROC curve that the predictive model had potential predictive discrimination and accuracy (Total set AUC = 0.680, Train set AUC = 0.679, Test set AUC = 0.680). **(D)** Calibration curve of the nomogram. UWES, Utrecht Work Engagement Scale; DCA, decision curve analysis; ROC, receiver operating characteristic; AUC, area under the curve.

At last, we drew a nomogram based on 7 variables above (age, gender, ethnicity, major, grade, native place, learning environment of your schools) as a method to predict low UWES probability (Figure 4A). With a higher score in the nomogram comes a lower UWES probability, thus a lower engagement in study. The accurate scores of each variable were listed in Table 3. Moreover, Table 4 listed how nomogram scores demonstrated low UWES probabilities quantitatively. After constructing the nomogram, we then used ROC and calibration curves to assess the internal validation of this nomogram. The DCA diagram showed that the medical students had better net benefits when the threshold probability was >0.2 (Figure 4B). What’s more, the ROC curve showed that the nomogram had predictive discrimination and accuracy (AUC) = 0.680 (Figure 4C). Besides that, the AUCs of the train set and the test set were almost identical, indicating that the model had a good predictive function. Moreover, calibration curve was excellent (Figure 4D), which proved that the predictive UWES scores corresponded with the actual ones.

**Table 3:** Nomogram scores of each variable.

Variables	Scores
Gender	
Female	9
Male	0
Age	
16–20	22
21–25	18
26–40	0
Ethnicity	
Minority	5
Ethnicity Han	0
Major	
Nursing	39
Preventive medicine	31
Preclinical medicine	18
Clinical medicine	15
Stomatology	0
Grade	
Grade 3	17
Grade 4	14
Grade 2	13
Grade 1	6
Graduate	6
Grade 5	0
Native place	
Municipality	19
Country	15
Town	13
Village	10
Provincial capital	1
Prefecture city	0
Learning environment of your schools	
Bad	100
Common	97
Terrible	83
Good	57
Excellent	0

**Table 4:** Nomogram scores and low UWES probabilities.

Low UWES probability	Scores
0.8	189
0.7	162
0.6	139
0.5	118
0.4	98
0.3	75
0.2	48
0.1	7

UWES, Utrecht Work Engagement Scale.

### Step 2: validation with JHLES

**Figure 5:**
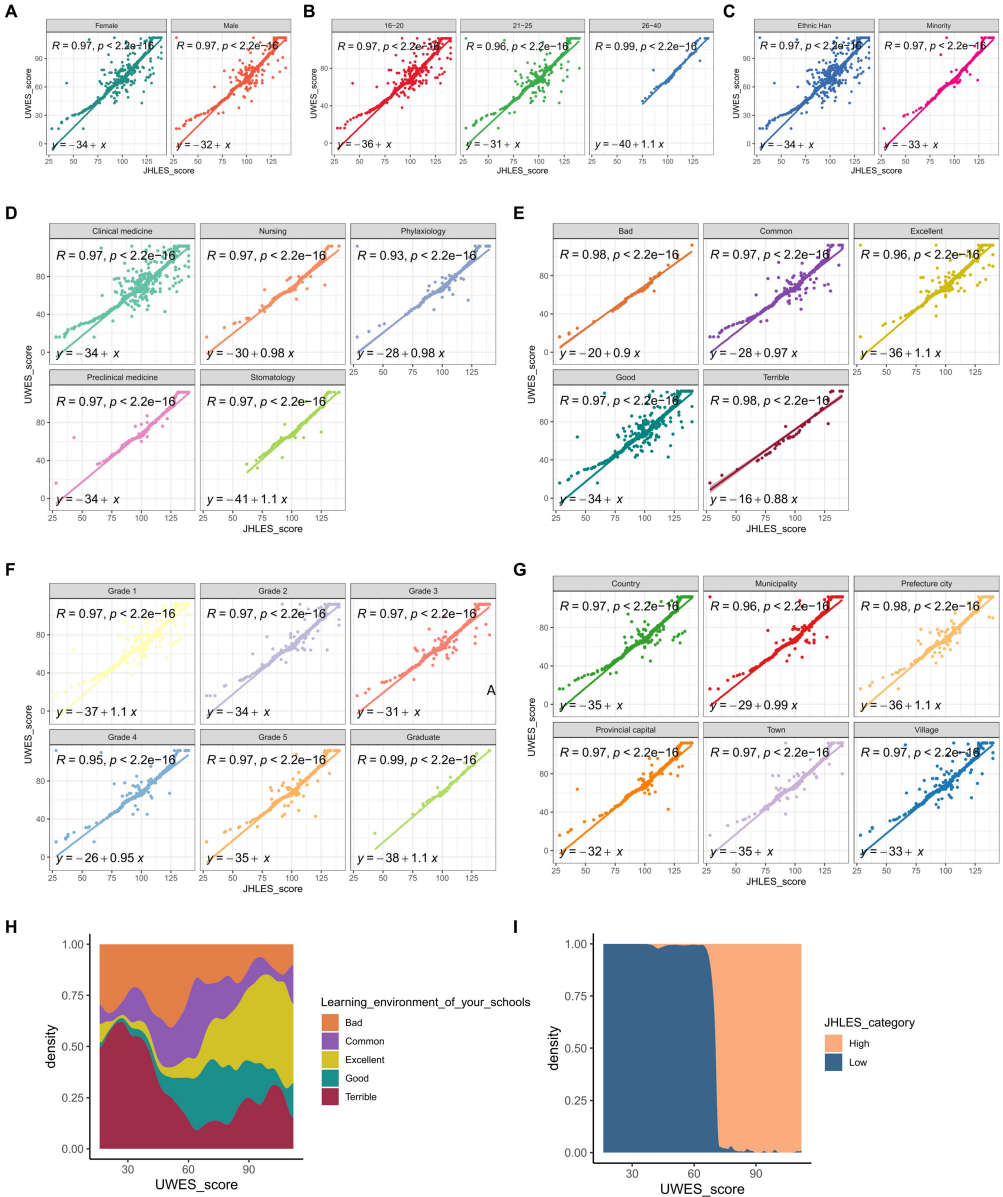
Validation of our study result with JHLES through correlation analysis and univariate liner regression analysis. The correlation analysis was performed across various subgroups: **(A)** gender, **(B)** age, **(C)** ethnicity, **(D)** major, **(E)** learning environment of your schools, **(F)** grade, and **(G)** native place. In all the subgroups, the result indicated that JHLES scores were positively correlated with UWES scores, with *p* < 0.001. **(H)** The density plot of the learning environment categories showed that students who rated their learning environment more favorably tended to have higher UWES scores. **(I)** The density plot for JHLES categories indicated that students with high JHLES scores consistently had high UWES scores, which aligns with the findings from the learning environment category density plot. UWES, Utrecht Work Engagement Scale; JHLES, Johns Hopkins Learning Environment Scale.

Because of the limitations of our method to assess student’s learning environment, as it relies on students’ subjective perceptions of the learning environment, we further conducted validation analysis. With JHLES, we quantitatively assessed medical students’ perception of learning environment. Then the univariate linear regression analysis was conducted, indicating that JHLES scores were significantly correlated with UWES scores in all subgroups (gender, age, ethnicity, major, learning environment of your schools, grade, and native place) (Figures 5A–G). Besides, the density plot of the learning environment categories showed that students who rated their learning environment more favorably tended to have higher UWES scores (Figure 5H). The density plot for JHLES categories indicated that students with high JHLES scores consistently had high UWES scores, which aligns with the findings from the learning environment category density plot (Figure 5I). All results could validate our result from Step 1 that a higher level of learning environment of medical school came with a higher level of medical students’ study engagement.

## Discussion

Having a high level of study engagement can help students to maintain academic satisfaction, improve physical and mental health and prevent dropping out of school (31, 32). For medical students, evidence has shown that although they are in higher level of wellbeing than their peers, they end up more distressed after graduation (33), which calls for an urgent need for improving medical students learning experience. However, the factors that influence the study engagement are not clear enough now.

In our study, we reached the conclusion that a better learning environment serves as a protective factor for enhancing study engagement among medical students. This finding is against some of the prior researches that has explored the relationship between learning environments and student engagement. For example, Hopper et al. compared student engagement across three different campuses, each offering diverse learning environments, and found no statistically significant differences in their levels of engagement (20). However, Klem and Connell indicated that study engagement acts as a mediating factor between students’ perceptions of the school environment and their academic performances (21). Furthermore, Van Ryzin et al. has demonstrated that students’ perceptions of support from their peers and teachers play a crucial role in enhancing learning engagement (22). These findings align closely with our conclusion, highlighting the importance of fostering supportive and conducive learning environments to promote student engagement and academic success.

**Figure 6:**
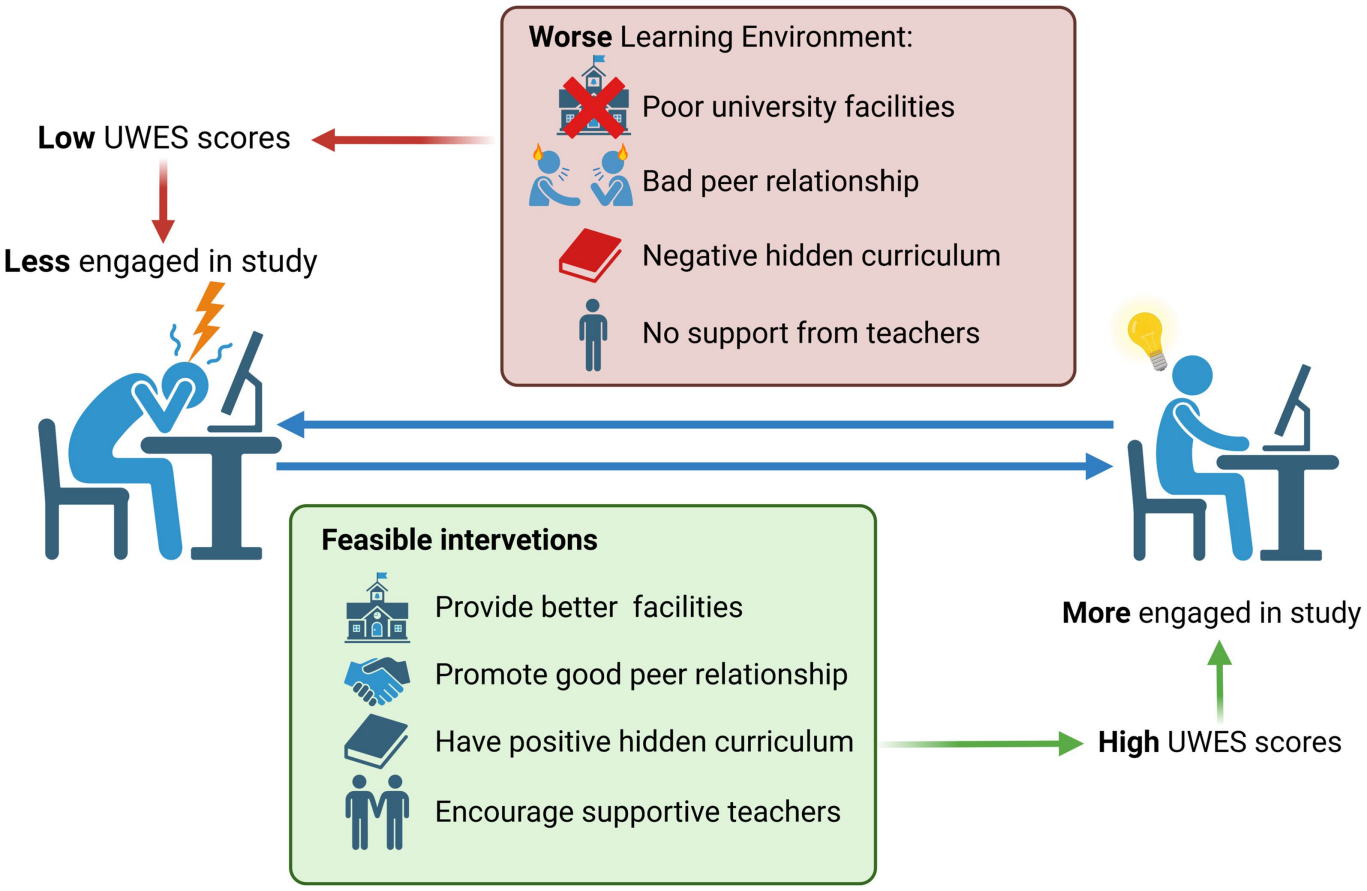
Schematic diagram. Created with BioRender.com. UWES, Utrecht Work Engagement Scale.

Recently, there has been a concerning and alarming phenomenon where the learning environment in medical schools has been linked to poor wellbeing among medical students (33, 34). Learning environment was made up of physical, social and psychological components (11), so many factors contributed to it. First, according to a previous research, only half of the students were satisfied with the physical learning environment, like academic-related facilities, interactive sessions or recreational facilities, provided by medical schools (35). Therefore, the improvement of learning facilities, which gave rise to a better learning environment, was a good choice to lift the quality of learning environment. For example, Belfi et al. found that virtual environment could help medical students to learn better introductory radiology, be more engaged with the class and feel more prepared for future clinical careers (36). Undergoing medical education was never an easy thing, because medical students had to deal with huge amounts of texts and graphs. Having a virtual learning environment helped medical students to review their work anytime and anywhere. Moreover, some abstract subjects like anatomy could provide medical students with a 3D model with the help of certain app, consolidating their knowledge. Second, the social component of the learning environment came from their peers. Peer support played an positive role in influencing study engagement (37, 38), because it could improve students’ self-efficacy (39). Self efficacy could be defined as ‘an individual belief in one’s capabilities to organize and execute the courses of action required in producing given attainments (40), and students with higher self-efficacy showed better engagement in study activities (41). In the university, which served as the rehearsal of society, the relationship between students was both close and subtle, because they lived all together in the university while competing with each other. Medical schools should promote good peer relationship between medical students and encourage students to either gain or give positive support to their peers. For example, certain curriculum, which was themed by maintaining good peer relationship, could be conducted in lower grade students, because they needed to lay a solid foundation with their new peers and they had less academic stress at that time. At last, psychological learning environment, which was the most important one, came from multiple aspects. The hidden curriculum, which was conceptualized as implicit or unintended influences that reflect certain culture, moral judgments, values and behaviors (42), was instilled into each medical students through social mechanisms (43). After observing the “role model” who might perform contradict to what medical students had learned in their professionalism curriculum, some medical students just lost the sense of “who they really are” and were cynical as well (44, 45). Taking the hidden curriculum into account was important for medical school when considering medical education. Medical schools should deliberate on the hidden curriculum that medical students encountered, train clinical professors to be good “role model” and wipe out the gap between hidden curriculum and professionalism curriculum. Moreover, the support from teachers also played an important part in providing a learning environment that contributed to study engagement. Perceived teacher autonomy support was associated with high level of students self-efficacy (46), which was a protective factor of high study engagement. In the context of university, where supervision from teachers was less than that from teachers in the high schools, it seemed that raising the support from teachers was a good choice for providing a good learning environment. Maybe an appraisal system could be built in universities, where students could evaluate how supportive each teacher was, and some incentives such as subsidies were accessible to the teachers with higher rank in support. Taken together, we summarized the major points of our study in a schematic diagram in Figure 6.

Our study concluded that the better the learning environment in your school was, the better the study engagement a medical student had. And we provide a theoretical model for predicting their study engagement with several variables including their perception of the learning environment. Introducing some necessary intervention to improve student’s study engagement in the aspect of learning environment was indispensable, and these interventions could be applied in the physical, social and psychological aspects. Although we only focused on the medical students in some centers in China, the result of our study can be applied to medical students in other centers in China or students in other majors by using analogy. For instance, comparing the study engagement and learning environments of medical students in China with those in other regions offers insights into how cultural factors influence medical education and student engagement across different cultural contexts. Moreover, our findings can provide guidance for educational policies and practices in countries confronting challenges similar to those in medical education and student engagement as observed in China. Additionally, researchers in other regions can replicate our study methodology to assess study engagement and learning environments in their own contexts, facilitating cross-validation of findings.

Some limitations in this study cannot be ignored. Firstly, although the number of questionnaires we collected was relatively substantial, it is essential to recognize that it only provides a snapshot of the situation among a portion of medical students in China. Furthermore, it’s important to note that all the participants were from medical schools located in modern cities. Although China boasts the largest medical education system worldwide (23), China ranks among the countries with the lowest ratio of medical schools per million inhabitants (24). The unique dynamics of China’s healthcare system, characterized by a high patient population and a relatively small number of doctors, necessitates a large and robust healthcare workforce (25). Given the unique circumstances prevailing in our study context, markedly different from those observed in developed countries, careful consideration is warranted when applying the conclusions of our study to medical students in other regions. Secondly, although we utilized a seven-pointed Likert scale to quantify medical students’ study engagement, the answers from them were still subjective, crippling the authenticity of the data. Thirdly, recalling bias occurred during students’ answering the questionnaire. Furthermore, in this study, we focused on a broad categorization to capture general trends, which meant that our method for assessing the learning environment was inherently subjective. While this approach has been used in several past studies (17, 47) and we also validated our result with JHLES, more detailed research is necessary to deepen our understanding of how the learning environment impacts study engagement among medical students.

## Conclusion

We concluded that learning environment of school was an independent factor of medical student’s study engagement. A higher level of learning environment of medical school came with a higher level of medical students’ study engagement. The construction of nomogram could serve as a helper for the educators or researchers to estimate medical students’ study engagement depending on their perception of learning environment. Some interventions of improving learning environment in medical schools were in need to promote study engagement.

## Data availability statement

The original contributions presented in the study are included in the article/Supplementary material, further inquiries can be directed to the corresponding author.

## Ethics statement

The study was approved by the Ethics Committee of the First Affiliated Hospital of Naval Medical University. The studies were conducted in accordance with the local legislation and institutional requirements. The participants provided their written informed consent to participate in this study.

## Author contributions

RH: Writing – review & editing, Writing – original draft, Visualization, Validation, Supervision, Software, Resources, Project administration, Methodology, Investigation, Formal analysis, Data curation, Conceptualization. YuL: Writing – review & editing, Writing – original draft, Visualization, Validation, Supervision, Software, Resources, Project administration, Methodology, Investigation, Formal analysis, Data curation, Conceptualization. MG: Writing – review & editing, Writing – original draft, Visualization, Validation, Supervision, Software, Resources, Project administration, Methodology, Investigation, Formal analysis, Data curation, Conceptualization. WZ: Writing – review & editing, Writing – original draft, Visualization, Validation, Supervision, Software, Resources, Project administration, Methodology, Investigation, Formal analysis, Data curation, Conceptualization. SX: Writing – review & editing, Writing – original draft, Visualization, Validation, Supervision, Software, Resources, Project administration, Methodology, Investigation, Formal analysis, Data curation, Conceptualization. JT: Writing – review & editing, Writing – original draft, Visualization, Validation, Supervision, Software, Resources, Project administration, Methodology, Investigation, Formal analysis, Data curation, Conceptualization. BL: Writing – review & editing, Writing – original draft, Visualization, Validation, Supervision, Software, Resources, Project administration, Methodology, Investigation, Formal analysis, Data curation, Conceptualization. YY: Writing – review & editing, Writing – original draft, Visualization, Validation, Supervision, Software, Resources, Project administration, Methodology, Investigation, Formal analysis, Data curation, Conceptualization. MJ: Writing – review & editing, Writing – original draft, Visualization, Validation, Supervision, Software, Resources, Project administration, Methodology, Investigation, Formal analysis, Data curation, Conceptualization. WQ: Writing – review & editing, Writing – original draft, Visualization, Validation, Supervision, Software, Resources, Project administration, Methodology, Investigation, Formal analysis, Data curation, Conceptualization. ZL: Writing – review & editing, Writing – original draft, Visualization, Validation, Supervision, Software, Resources, Project administration, Methodology, Investigation, Formal analysis, Data curation, Conceptualization. HM: Writing – review & editing, Writing – original draft, Visualization, Validation, Supervision, Software, Resources, Project administration, Methodology, Investigation, Formal analysis, Data curation, Conceptualization. XWu: Writing – review & editing, Writing – original draft, Visualization, Validation, Supervision, Software, Resources, Project administration, Methodology, Investigation, Formal analysis, Data curation, Conceptualization. HY: Writing – review & editing, Writing – original draft, Visualization, Validation, Supervision, Software, Resources, Project administration, Methodology, Investigation, Formal analysis, Data curation, Conceptualization. XL: Writing – review & editing, Writing – original draft, Visualization, Validation, Supervision, Software, Resources, Project administration, Methodology, Investigation, Formal analysis, Data curation, Conceptualization. CZ: Writing – review & editing, Writing – original draft, Visualization, Validation, Supervision, Software, Resources, Project administration, Methodology, Investigation, Formal analysis, Data curation, Conceptualization. ED: Writing – review & editing, Writing – original draft, Visualization, Validation, Supervision, Software, Resources, Project administration, Methodology, Investigation, Formal analysis, Data curation, Conceptualization. QL: Writing – review & editing, Writing – original draft, Visualization, Validation, Supervision, Software, Resources, Project administration, Methodology, Investigation, Formal analysis, Data curation, Conceptualization. ZH: Writing – review & editing, Writing – original draft, Visualization, Validation, Supervision, Software, Resources, Project administration, Methodology, Investigation, Formal analysis, Data curation, Conceptualization. ML: Writing – review & editing, Writing – original draft, Visualization, Validation, Supervision, Software, Resources, Project administration, Methodology, Investigation, Formal analysis, Data curation, Conceptualization. XWa: Writing – review & editing, Writing – original draft, Visualization, Validation, Supervision, Software, Resources, Project administration, Methodology, Investigation, Formal analysis, Data curation, Conceptualization. YW: Writing – review & editing, Writing – original draft, Visualization, Validation, Supervision, Software, Resources, Project administration, Methodology, Investigation, Formal analysis, Data curation, Conceptualization. WC: Writing – review & editing, Writing – original draft, Visualization, Validation, Supervision, Software, Resources, Project administration, Methodology, Investigation, Formal analysis, Data curation, Conceptualization. YiL: Writing – review & editing, Writing – original draft, Visualization, Validation, Supervision, Software, Resources, Project administration, Methodology, Investigation, Formal analysis, Data curation, Conceptualization. JZ: Writing – review & editing, Writing – original draft, Visualization, Validation, Supervision, Software, Resources, Project administration, Methodology, Investigation, Funding acquisition, Formal analysis, Data curation, Conceptualization. SJ: Writing – review & editing, Writing – original draft, Visualization, Validation, Supervision, Software, Resources, Project administration, Methodology, Investigation, Funding acquisition, Formal analysis, Data curation, Conceptualization.

## Footnotes

^1^ https://www.wjx.cn/
